# Importance of Early Detection of Wire Syndrome: A Case Series Illustrating the Main Stages of the Clinical Gradient

**DOI:** 10.3390/clinpract13050098

**Published:** 2023-09-11

**Authors:** Carole Charavet, Nathan Israël, France Vives, Sophie-Myriam Dridi

**Affiliations:** 1 Département d’Orthodontie, Faculté de Chirurgie-Dentaire, Université Côte d’Azur, 06300 Nice, France; nath_925@hotmail.fr; 2 UEC d’Orthodontie, Institut de Médecine Bucco-Dentaire, Centre Hospitalier Universitaire de Nice, 06300 Nice, France; 3 Laboratoire de Microbiologie Orale, Immunothérapie et Santé (MICORALIS) UPR7354, Faculté de Chirurgie-Dentaire, Université Côte d’Azur, 06300 Nice, France; 4 Pratique Privée, 75116 Vitrolles, France; f.vives@hotmail.fr; 5 Département de Parodontologie, Faculté de Chirurgie-Dentaire, Université Côte d’Azur, 06300 Nice, France; dr.sm.dridi@free.fr; 6 UF de Parodontologie, Institut de Médecine Bucco-Dentaire, Centre Hospitalier Universitaire de Nice, 06300 Nice, France

**Keywords:** wire syndrome, bonded retainer, fixed retainer, orthodontic retainer, unexpected movement, unwanted movement

## Abstract

(1) Context and Objective: Wire syndrome (WS) refers to dental displacements which can be qualified as aberrant, unexpected, unexplained, or excessive of teeth still contained by an intact orthodontic retainer wire without detachment or fracture, leading to evolving aesthetic and/or functional consequences, both dental and periodontal. The clinical diagnosis of WS in severe cases is often easy. On the other hand, emerging cases must be detected early to stop this evolutionary process as soon as possible, as well as to effectively manage unwanted dental displacements and associated dento-periodontal tissue repercussions. The aim of this retrospective study was to understand the challenges and importance of early diagnosis, highlight the clinical gradient of WS, and clarify the key elements of diagnosis for many practitioners confronted with this type of problem. (2) Materials and Methods: Three cases of increasing complexity were described: the history of wire syndrome, a description of the key elements of its diagnosis, and the final diagnosis itself. (3) Results: Different types and locations of wire syndrome have been observed, from early form to terminal wire syndrome. The three main stages of the clinical gradient are described as follows. In the first case, wire syndrome starting on tooth 41, called the “X-effect” type, was suspected. X-effect wire syndrome on 21, X-effect wire syndrome on 41, and Twist-effect wire syndrome on 33 were diagnosed in the second case, which can be classified as an intermediate case. In the extreme clinical situation of the last case, severe and terminal wire syndrome on tooth 41, the X-effect type, was observed. (4) Conclusions: This case series presents the main stages of the clinical gradient of WS. Although in the case of early WS it is very difficult to identify and/or differentiate it from movements related to a classical relapse phenomenon, the diagnosis of terminal WS is very easy. The challenge for the practitioner is therefore to detect WS as early as possible to stop the iatrogenic process and propose a personalized treatment depending on the severity of clinical signs. The earlier WS is detected, the less invasive the treatment.

## 1. Introduction

In 2007, for the first time, Katsaros et al. [1] published a case report of an unreported complication associated with bonded orthodontic retainers under the title “unexpected complications of bonded mandibular lingual retainers”. Subsequently, other authors have also observed the same complication, which has been given several different names. For example, Abudiak et al. (2011) [2] and Pazera et al. (2012) [3] described “a complication with orthodontic fixed retainer” and “a severe complication of a bonded mandibular lingual retainer”, respectively. In 2015 in France, Roussarie et al. [4,5] were the first to refer to this phenomenon as “Syndrome du Fil” (SF), translated into English as “wire syndrome (WS)” (Table 1). 

The first systematic review on wire syndrome (WS) was published by Charavet et al. [21] in 2022, defining its prevalence and associated characteristics. This review involved an electronic search strategy on four databases supplemented by a manual search. All prospective and retrospective clinical studies, including case reports and case series written in English or French, with a clear description, detection, or management of WS, were included. Out of 1891 results, 20 articles were selected. They were published between 2007 and 2021, mostly in the form of series or case reports; the overall risk of bias was considered high. The prevalence of WS varied from 1.1% (10 out of the 3500 patients studied) to 27% (18 out of the 163 patients studied). The orthodontic retainer was found to be intact, without fractures or debonding, except in severe cases where it was altered at the end of the process. WS was found in patients of all ages, did not depend on gender, and most often developed in the first five years following the placement of fixed retainers. It is most commonly found in the mandible where the canines are affected more than the incisors. Although most of the retainer wires involved in WS are round, multi-strand, twisted stainless steel wires, all types of wires can be involved, including flat, braided chains. The results of the systematic review led to a proposed clinical definition of WS. This syndrome refers to dental displacements, which can be qualified as aberrant, unexpected, unexplained, or excessive on teeth, still contained by an intact orthodontic retainer wire without detachment or fracture, leading to evolving aesthetic and/or functional consequences, both dental and periodontal. 

Once WS occurs, dental and periodontal consequences, which are initially minor, progressively worsen over time. However, in the case of early WS, it is very difficult to identify and/or differentiate it from movements related to a classical relapse phenomenon. Therefore, the practitioner’s challenge is to diagnose WS as early as possible to stop the process and propose a personalized treatment according to the severity of the clinical signs. The aim of this article was also to illustrate three clinical cases of increasing severity to demonstrate the importance of early detection, highlight the clinical gradient of WS, and clarify the key elements of the diagnosis.

## 2. Materials and Methods

Registration: This study has been approved/registered by the Data Protection Officer of the Digital Innovation and Information Systems Department on the Nice University Hospital’s data processing register (reference 2023—EI 548).

Patient selection: Three clinical cases of wire syndrome of increasing severity in patients who were admitted to the orthodontic department of the University Hospital of Nice were retrospectively included. They presented early wire syndrome, intermediate wire syndrome, and severe wire syndrome.

Data collection: A full set of photographs were taken from the following perspectives: frontal view, different lateral views, and different occlusal views.

Data analysis: The description and analysis of the pictures mainly focused on the detection of a wire syndrome; other elements were not considered.

## 3. Results

### 3.1. Early Wire Syndrome

**Figure 1:**
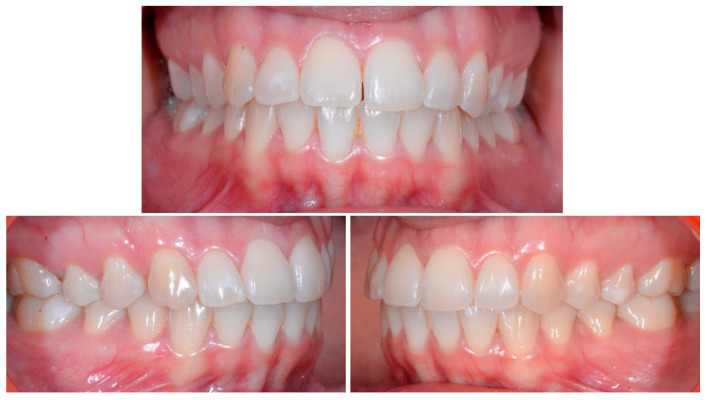
Early wire syndrome. Frontal and lateral views.

A 22-year-old female patient in good health had a consultation because she was concerned about the “root prominence” of tooth 41. She wore a mandibular retainer wire when she was 16 years old at the end of her orthodontic treatment. She has good oral hygiene, despite the presence of tartar between 31 and 41, and a right and left Class I with a slight deviation of the midlines (Figure 1). 

**Figure 2:**
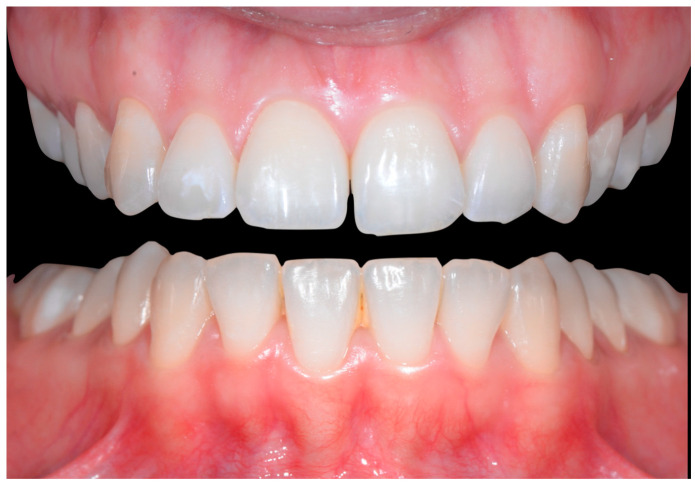
Early wire syndrome. Frontal views.

In Figure 2, the following can be observed: a difference in gingival margin height between 41 and 31, a difference in height of the incisal edges of 41 compared to the adjacent teeth, and the onset of gingival recession on 41 associated with the visibility of a vestibular arch corresponding to its root. 

**Figure 3:**
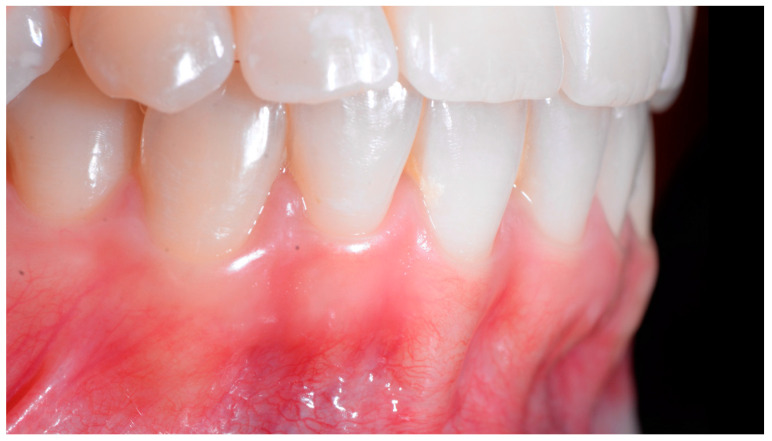
Early wire syndrome. Lateral view.

In the lateral view (Figure 3), the prominence of the root of 41 was confirmed. 

**Figure 4:**
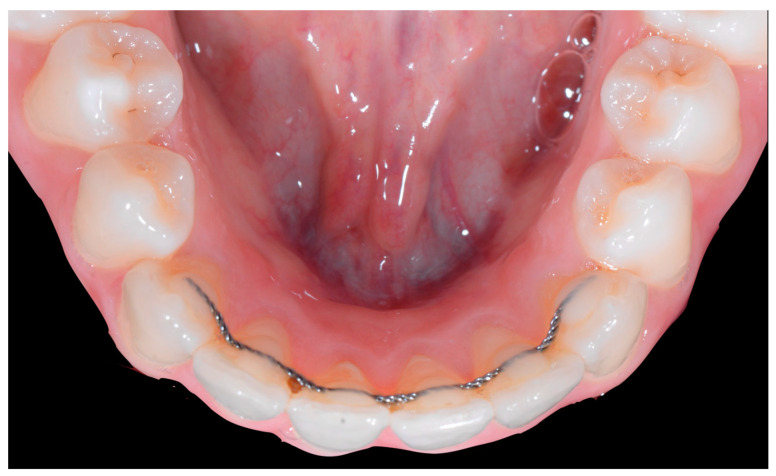
Early wire syndrome. Occlusal view.

Occlusal views (Figure 4) confirmed the presence of a mandibular retainer which appears intact, without fracture or debonding, along its entire length. In addition, the vestibular surface of 41 appeared to have a difference in visibility from the adjacent teeth. Based on these clinical findings, wire syndrome starting on tooth 41, called the “X-effect” type, was suspected.

### 3.2. Intermediate Wire Syndrome

**Figure 5:**
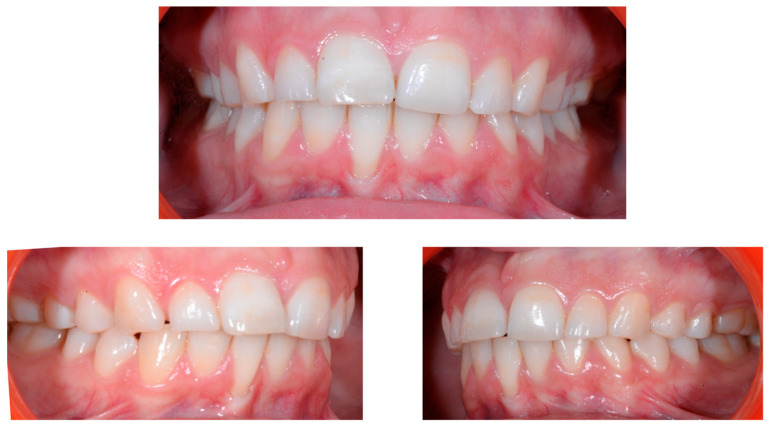
Intermediate wire syndrome. Frontal and lateral views.

A 26-year-old female patient was referred by her general dentist for suspected wire syndrome. Orthodontic treatment had been performed 10 years previously, and bonded restorations had been fitted at the end of the treatment. The patient mentioned several episodes of breakage/adhesion, without further details. She has good oral hygiene and a right and left Class I (Figure 5).

**Figure 6:**
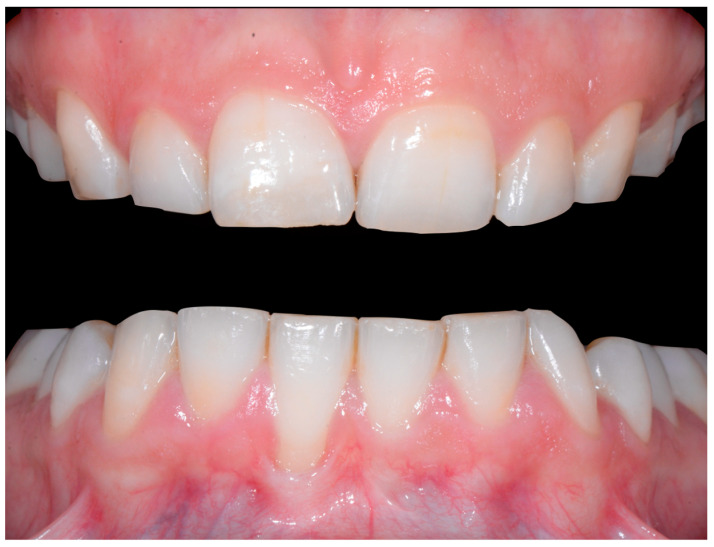
Intermediate wire syndrome. Frontal view.

In Figure 6, 11 and 21 show a difference in incisal edge height and gingival margin. Tooth 41 shows gingival recession to the muco-gingival junction (Cairo’s RT1) with root visibility. Tooth 33 had a significant lingual tilt (coronal–lingual torque), not symmetrical to tooth 43.

**Figure 7:**
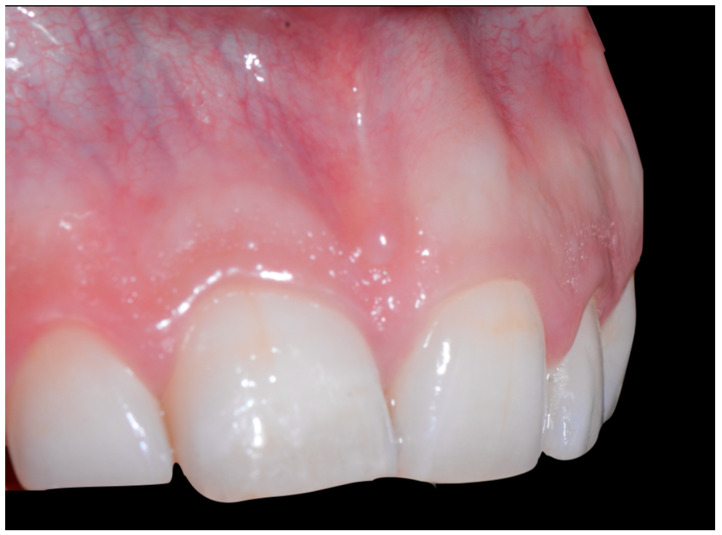
Intermediate wire syndrome. Lateral view.

**Figure 8:**
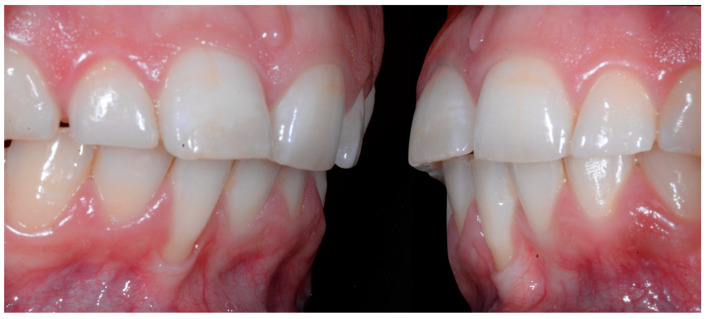
Intermediate wire syndrome. Lateral views.

The root of tooth 21 is visible through the gingiva (Figure 7). Figure 8 shows the extent of gingival recession on tooth 41. 

**Figure 9:**
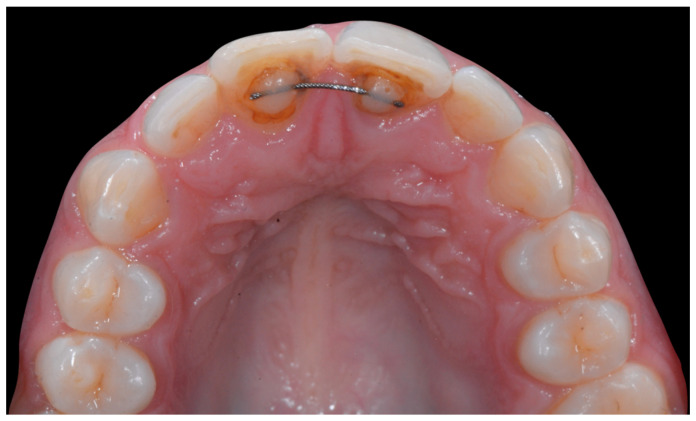
Intermediate wire syndrome. Occlusal view.

**Figure 10:**
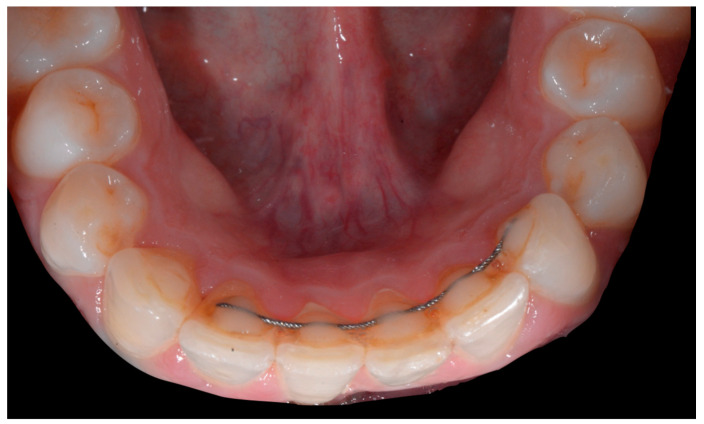
Intermediate wire syndrome. Occlusal view.

The occlusal views provide additional relevant information (Figure 9 and Figure 10). A maxillary retainer was present on 11 and 21 only and a difference in visibility of the vestibular surfaces (differential torque) on these same teeth was noted. 

In the mandible, the retainer was broken distal to 42 and, despite being intact on 33, this tooth had increased visibility of its vestibular surface compared to its contralateral tooth (differential torque). Finally, teeth 31 and 41 also showed a difference in the visibility of their vestibular surfaces (differential torque). Ultimately, the patient was diagnosed with an X-effect wire syndrome on 21, an X-effect wire syndrome on 41, and a Twist-effect wire syndrome on 33. 

### 3.3. Severe Wire Syndrome

**Figure 11:**
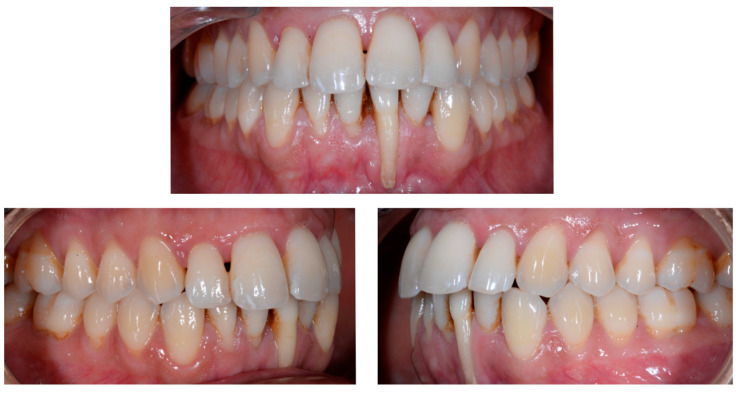
Severe wire syndrome. Frontal and lateral views.

A 40-year-old patient presented with discomfort in tooth 31, citing past orthodontic treatment. As shown in Figure 11, the patient was in Class I and had poor oral hygiene associated with the presence of calculus in the incisivo-canine region. The root of 31, visible to its apex, was out of the bone and associated with severe gingival recession (Cairo RT2). Teeth 41 and 42 also showed gingival recession (Cairo RT2 and RT1). 

**Figure 12:**
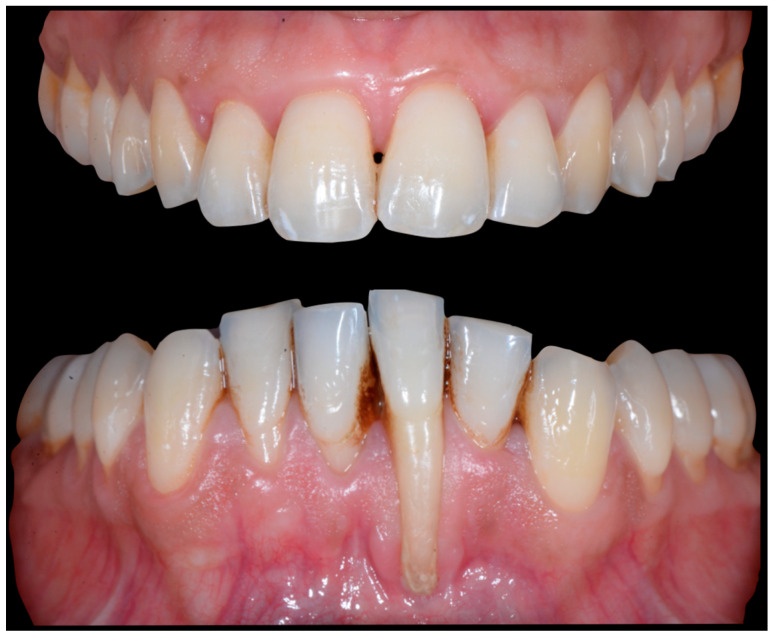
Severe wire syndrome. Frontal view.

**Figure 13:**
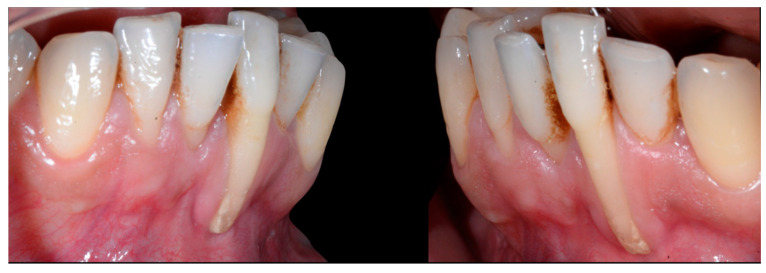
Severe wire syndrome. Lateral views.

Figure 12 and Figure 13 show a difference in the height of the free edges of the mandibular incisors and the extent of root visibility of 31. 

**Figure 14:**
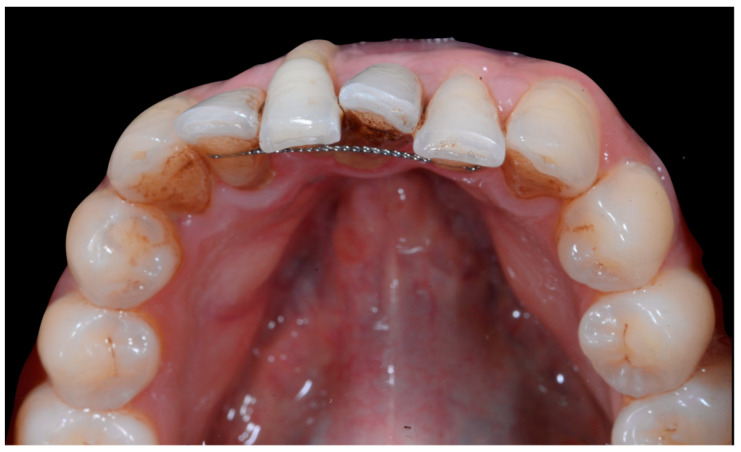
Severe wire syndrome. Occlusal view.

No retainer was present in the maxilla, only a residual mandibular retainer, still bonded to 32 and 42, was visible (Figure 14), as well as incisal crowding and a difference in the visibility of the buccal and root surfaces of 41 compared to the contralaterals. In this extreme clinical situation, a severe and terminal wire syndrome on tooth 41, the “X-effect” type, was observed. 

## 4. Discussion

The movements caused by WS do not correspond to a relapse or a physiological process [21]; the effects of WS on teeth are not correlated with the position of teeth before orthodontic treatment nor with their position at the time of the debonding of an orthodontic appliance. WS can therefore be considered as a new malocclusion observed after the placement of a fixed, bonded, post-orthodontic retainer [21]. The aim of this article was to show, via three clinical cases representing three main situations of WS of increasing complexity, the relevance of the early diagnosis of this syndrome.

**Figure 15:**
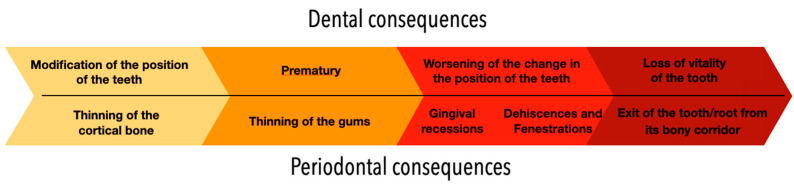
Summary of evolving dental and periodontal consequences of wire syndrome over time.

A systematic review by Charavet and al. [21] identified four main families of typical movements: a torque differential between two adjacent incisors called the “X-effect”, an opposite inclination between two contralateral canines called the “Twist-effect”, an exaggerated torque on a tooth, or a non-specific complication (opening of space). These movements can also be associated with each other, and a variety of clinical manifestations can then be observed. Once WS occurs, the consequences for hard and soft tissues, which are initially minor, progressively worsen over time (Figure 15). However, this systematic review was not able to elucidate the circumstances of WS emergence and its progression. Therefore, it seemed important to clarify these points, based on our clinical impressions and expertise.

Although it is reasonable to assume that tissue damage occurs from the initiation of FS, clinical manifestations start to be visible when the disease process results in root displacement. We can therefore propose a clinical gradient with dental and periodontal consequences varying from a minor problem to the exposure of the root to the apex of the affected tooth (Figure 4). The three clinical situations described in this article illustrate the main stages of this gradient.

When the displacement due to wire syndrome is minimal, the result is a slight displacement of the affected tooth, with the maintenance of contact points but the loss of tooth neck (cervical margin) alignment and the appearance of discrete gingival recession (Figure 1). In the lateral view, the displacement of the tooth is visible and can be objectified via the digital palpation of the root. In the occlusal view, the incisal edges are no longer completely aligned. Over time, the more the root becomes displaced, the thinner the gingiva becomes and the more the attachment worsens, first on the entire face of the tooth (vestibular or lingual) and then in the interdental area. Clinically, this results in increasingly wide and deep gingival recessions and the loss of tooth alignment (Figure 2). Eventually, interdental spaces appear and the displacement of the roots from their sockets is major. Therefore, in the presence of very large tooth displacements (end of the WS process), the orthodontic retainer becomes detached or breaks (Figure 3).

In the present case of severe WS, generalized moderate periodontitis was diagnosed, except on the mandibular incisors, which showed a more severe attachment loss. This can be explained by the presence of the WS, which aggravates the pathological process. The presence of bacterial and calculus deposits on the incisors was certainly important, but it alone cannot explain the severity of tissue destruction, particularly on 31. This case highlights the problem of the multi-factorial etiology of WS.

Concerning the risk factors associated with the development of WS, several authors point to inconsistent results, except for the presence of onycophagy parafunction. Furthermore, etiological hypotheses have been associated with the practitioner (bonding of a non-passive retainer wire, deformation of the wire during bonding, etc.) or with the wire (deformation of the wire, instability of its mechanical properties, etc.), and thus associated preventive measures have been proposed [21]. Finally, the complexity of the treatments, often multidisciplinary, is proportional to the severity of the clinical situations [21]. Currently, the management of wire syndrome is based on the clinical common sense of clinicians because there is no scientific-supported consensus.

The present case series has some limitations. First, our hypotheses are not supported by scientific evidence. However, the present case series constitutes a starting point for the development of cross-sectional observation studies. For ethical reasons, it seems difficult to perform prospective trials of exposed/unexposed cohorts. It is not possible to avoid treating patients with WS (in case of a control group) with the unique aim of identifying the risk factors associated with this syndrome. Furthermore, we focused solely on the clinical criteria and did not address the therapeutic aspect. In fact, we also think it interesting to begin by establishing the foundations of a diagnostic approach based on relevant diagnostic criteria before proposing a potentially multidisciplinary treatment pathway. 

## 5. Conclusions

The identification of wire syndrome is recent and of increasing concern to clinicians, orthodontists, general practitioners, and periodontists. Indeed, while severe forms of wire syndrome are very easily detectable, early forms are more difficult to detect and can be confused with a classic orthodontic relapse. The stakes are high; early detection allows for immediate treatment and the application of appropriate therapy, thus preventing this iatrogenic process. However, additional clinical studies are necessary to clarify the etiopathogenic mechanisms of wire syndrome and its clinical impacts, ultimately allowing us to suggest effective preventive therapies.

## Figures and Tables

**Table 1:** Names of wire syndrome (WS) over time.

Authors	Date	Name
Katsaros et al. [1]	2007	Unexpected complications of bonded mandibular lingual retainers
Abudiak et al. [2]	2011	A complication with orthodontic fixed retainers
Renkema et al. [6]	2011	Unexpected posttreatment complications
Alessandri Bonetti et al. [7]	2012	Isolated-type recession defects with an abnormal buccolingual inclination
Pazera et al. [3]	2012	Severe complication of a bonded mandibular lingual retainer
Farret et al. [8]	2015	Extreme labial movement of the root
Roussarie et al. [4,5]	2015 and 2018	Syndrome du fil
Kučera et al. [9,10]	2016	Unexpected complications/X-effect, Twist-effect and non-specific complications
Laursen et al. [11]	2016	Complications after unintentional tooth displacement by active bonded retainers
Shaughnessy et al. [12]	2016	Inadvertent tooth movement with fixed lingual retainers
Wolf et al. [13]	2016	Undesired tooth movement
Egli et al. [14]	2017	Unexpected posttreatment changes
Jacobs et al. [15]	2017	Single tooth torque problems
Beitlitum et al. [16]	2020	Unwanted effects such as inadvertent tooth movement and torque changes
Kim et al. [17]	2020	Unexpected tooth movements
Klaus et al. [18]	2020	Unwanted tooth movements
Knaup et al. [19]	2021	Side effects of twistflex retainers
Singh et al. [20]	2021	Extreme complication of a fixed mandibular lingual retainer
